# Supplementary material for: Assessment of deep neural networks for the diagnosis of benign and malignant skin neoplasms in comparison with dermatologists: A retrospective validation study
Source: PLoS Med. 2020 Nov 25;17(11):e1003381. doi: 10.1371/journal.pmed.1003381 (PMC7688128; doi:10.1371/journal.pmed.1003381)
Supplement: S1 Text — (DOCX) [file pmed.1003381.s002.docx]

**S1 Text. Supplementary Methods and Results**

**METHODS**

**Algorithm**

To evaluate the generalizability of neural networks for skin neoplasm classification, an algorithm developed in a previous study [1] was tested again without modification in this study. The web-DEMO of the algorithm has been made publicly accessible (http://rcnn.modelderm.com) to facilitate scientific communications. The algorithm was trained not only with hospital archives, but also with archives generated with the assistance of a region-based convolutional neural network (RCNN) [2, 3] to reduce false positives. In total, our algorithm was trained with 1,106,886 image crops.

The algorithm has three parts, which were trained separately: (1) a blob detector (faster RCNN), (2) a fine image selector (CNN), and (3) a disease classifier part (CNN). We used the three-stage approach because it has several advantages over the single-stage approach: 1) Efficient annotation; By dividing the scope of the problem, the training dataset required for each problem can be obtained separately. 2) Improved explainability; The causes of incorrect predictions can be analyzed based on whether such predictions resulted from (a) incorrect blob detection, (b) inadequate image quality, or (c) incorrect prediction of the disease classifier. 3) Dealing with imbalance; There are many general objects and trivial skin lesions that lead to imbalance in the training dataset. By dividing the stages, the final disease classifier can be trained with a less imbalanced training dataset.

*Training of Blob Detector*

The blob detector was trained with 21,421 images of nodular disorders obtained from the Asan Medical Center and 103,627 general object images from ImageNet. When we built a dataset in a previous study [4], we annotated the lesions’ locations to crop the lesions of interest. The blob detector was trained using faster-RCNN (backbone = VGG-16; <https://github.com/rbgirshick/py-faster-rcnn>; NMS_THRESHOLD=0.3 and CONF_THRESHOLD=0.1). The blob detector can suggest possible lesions of interest from unprocessed clinical images.

*Training of Fine Image Selector*

After training the blob detector, it was deployed on 182,348 unprocessed images from the Asan Medical Center and websites, to generate numerous possible blobs. Based on the image findings, we manually classified these blobs as 1) 81,030 fine blobs, 2) 59,319 inadequate blobs, 3) 270,224 normal or nonspecific blobs, and 4) 152,493 general object blobs. The fine image selector (SE-ResNet-50) was trained to exclude inadequate lesions or general objects.

*Training of Disease Classifier*

After training the blob detector and fine image selector, we employed the blob detector and fine image selector using 182,348 unprocessed images from the Asan Medical Center and websites, to generate numerous lesions. The diagnoses of the generated lesions were tagged based on image findings. The disease classifier was trained with 721,749 image crops corresponding to 178 disease classes. We fine-tuned SENet and SE-ResNeXt-50 end-to-end, separately (learning_rate = 0.01, gamma = 0.1, weight_decay = 0.00001, mini_batch_size = 32, solver = SGD, momentum = 0.9, total_iteration = 90 epochs, and step_iteration = 30 epochs). The final output was obtained by arithmetically averaging the outputs of the two models.

**Quantifiable Differences between Training and Test Datasets**

*Training Dataset*

A total of 1,106,886 image crops which were generated from 182,348 unprocessed images were used for the training (178 disorders; Asian from the Asan dataset and various races from websites).

*Test Dataset (Severance Dataset)*

A total of 40,331 unprocessed images were used for the external testing (43 skin neoplasm, mainly Asian).

*Test Dataset (Edinburgh Dataset)*

A total of 1,300 image crops were used for another external testing (10 benign and malignant nodules, mainly White).

**Formulation of the Malignancy Output and Predefined Thresholds**

The malignancy output was defined as: “the sum of outputs of malignant disorders” + 0.2 × “the sum of outputs of premalignant disorders”. Premalignant lesions such as actinic keratosis (AK) require particular attention and close observation. Therefore, lesions highly suspected to be AK (AK output = 1.0) were considered and weighted because they would be otherwise neglected for the end user.

The physicians evaluated each lesion as “biopsy or not” / “malignant or not”. Similarly, the algorithm generated reports with two different values, corresponding to a high-sensitivity threshold and a high-specificity threshold. The high-sensitivity threshold was formerly [1] denoted as T_90_ in the previous study, and the high-specificity threshold as T_80_. These cut-off thresholds were defined as 90% or 80% sensitivity points for the validation dataset (images from 386 patients) obtained from the Asan Medical Center. All patients clinically suspected of 10 major tumorous disorders (the same tumors as those from the Edinburgh dataset) from January 1, 2018 to June 30, 2018, were initially included. After pathologic confirmation, the final validation dataset comprised of malignant tumors (81 patients), benign tumors (251 patients), and various other benign disorders (54 patients). The high-sensitivity threshold was defined as the threshold at which 90% sensitivity was obtained for the validation dataset (images from 386 patients), whereas the high-specificity threshold was defined as the threshold at which 80% sensitivity was obtained. The preliminary values for the high-sensitivity threshold and high-specificity threshold without adding “0.2 × premalignant” were 0.2278 and 0.4451, respectively.

We chose “0.2” as a weight value because “0.2” is close to the high-specificity threshold (=0.2278) for lesions very likely to be premalignant. For instance, lesions suspected to be AK with 100% certainty were given a weighted factor of 0.2, which gave a warning message to the user, although it is not reported as being malignant (0.2 < 0.2278). If not, the malignancy output for the AK lesion with 100% certainty would become zero, which would convey false reassurance. The final thresholds with a weighted calculation of 0.2 × premalignant were assessed with the validation dataset again; the values were determined to be 0.2545 and 0.4687, respectively.

**Conversion and Assessment of the Response of Clinicians**

Clinicians can provide Top1, Top2, and Top3 diagnoses but not the probability score. In contrast, the algorithm can provide Top1, Top2, and Top3 diagnoses along with the probability of each diagnosis. In addition, the malignancy output of the algorithm can be calculated with those probabilities. For the binary classification (malignancy or not), a ROC curve of the algorithm can be obtained because the algorithm provides probability scores (malignancy output). For the clinicians, in contrast, their results can only be drawn with three ‘dots’ because clinicians usually answer “Yes” or “No”, rather than provide a probability score.

The specific clinical diagnosis can be transformed to a binary decision (malignancy or not). For example, assume the following diagnoses were provided by clinicians:

First impression of clinician = Seborrheic keratosis

Second impression of clinician = Basal cell carcinoma

Third impression of clinician = Actinic keratosis.

Then, because the Top-n accuracy is defined as the probability of the true diagnosis (= malignancy) being one of the (n) number of responses, each response would correspond to the following in the question of ‘malignancy or not’:

Top-1 = Benign

Top-2 = Malignancy

Top-3 = Malignancy.

**RESULTS**

Herein, we describe the results of the multi-class classification. In this study, multi-class classification is used to predict the exact diagnosis, whereas binary classification is used to determine whether a certain lesion is cancerous or not. The multi-class classification was performed with Severance Dataset B and the Edinburgh dataset. For the multi-class test, all 178 outputs was used to reflect the real environments where various conditions should be considered.

**(A) Multi-class classification of 32 Skin Tumors using Severance B dataset**

For the multi-class classification test, we used AUC and Top-accuracy as evaluation metrics.

The calculation of Top accuracies of each class was performed with the images of Severance Dataset B (39,721 images from 10,315 cases, 32 disorders). We excluded 6 classes (angiofibroma, Café au lait macule, juvenile xanthogranuloma, milium, nevus spilus, and sebaceous hyperplasia) where the number of patients was less than 10, and we also excluded 5 classes (Spiz nevus, dermatofibrosarcoma protuberans, angiosarcoma, Kaposi’s sarcoma, and Merkel cell carcinoma) on whose data the algorithm was not trained. The macro-averaged mean Top-1,2,3 accuracies of the clinical diagnoses were 65.4±17.7%, 73.9±16.6%, and 74.7±16.6%, respectively, and those obtained using the algorithm were 42.6±20.7%, 56.1±22.8%, and 61.9±22.9%, respectively. The micro-averaged mean Top-1,2,3 accuracies of the clinical diagnoses were 68.2%, 77.5%, and 78.7% and those of the algorithm were 49.2%, 63.9%, and 71.2%, respectively.

The AUC of each disease class was calculated by transforming the multi-class problem into a binary-class problem using the one-versus-rest method. The mean AUC of 32 classes was 0.931±0.062 (mean±STD; S1 Fig and S1 Table).

**(B) Multi-class classification of 10 Skin Tumors using the Edinburgh dataset**

We performed an additional external validation with the Edinburgh dataset. The Edinburgh dataset consists primarily of data corresponding to white subjects, and the dataset, which includes images corresponding to 10 benign and malignant skin tumors is commercially available (S2 Table; https://licensing.edinburgh-innovations.ed.ac.uk/i/software/dermofit-image-library.html). In previous studies[4, 5], the Edinburgh dataset was used as a validation dataset.

For the multi-class classification, the macro-averaged mean Top-1,2,3 accuracies were 53.0%, 70.8%, and 77.6%, respectively, and the mean AUC of each class was 0.939±0.030 (mean±SD; S2 Fig and S2 Table).

**REFERENCES**

1. Han SS, Moon IJ, Lim W, Suh IS, Lee SY, Na J-I, et al. Keratinocytic Skin Cancer Detection on the Face Using Region-Based Convolutional Neural Network. JAMA dermatology. 2019. doi: 10.1001/jamadermatol.2019.3807.

2. Ren S, He K, Girshick R, Sun J, editors. Faster r-cnn: Towards real-time object detection with region proposal networks. Advances in neural information processing systems; 2015.

3. Han SS, Park GH, Lim W, Kim MS, Im Na J, Park I, et al. Deep neural networks show an equivalent and often superior performance to dermatologists in onychomycosis diagnosis: Automatic construction of onychomycosis datasets by region-based convolutional deep neural network. PloS one. 2018;13(1):e0191493.

4. Han SS, Kim MS, Lim W, Park GH, Park I, Chang SE. Classification of the Clinical Images for Benign and Malignant Cutaneous Tumors Using a Deep Learning Algorithm. Journal of Investigative Dermatology. 2018.

5. Esteva A, Kuprel B, Novoa RA, Ko J, Swetter SM, Blau HM, et al. Dermatologist-level classification of skin cancer with deep neural networks. Nature. 2017;542(7639):115-8. doi: 10.1038/nature21056. PubMed PMID: 28117445.
