# Supplementary figures and images for: Assessment of deep neural networks for the diagnosis of benign and malignant skin neoplasms in comparison with dermatologists: A retrospective validation study
Source: PLoS Med. 2020 Nov 25;17(11):e1003381. doi: 10.1371/journal.pmed.1003381 (PMC7688128; doi:10.1371/journal.pmed.1003381)

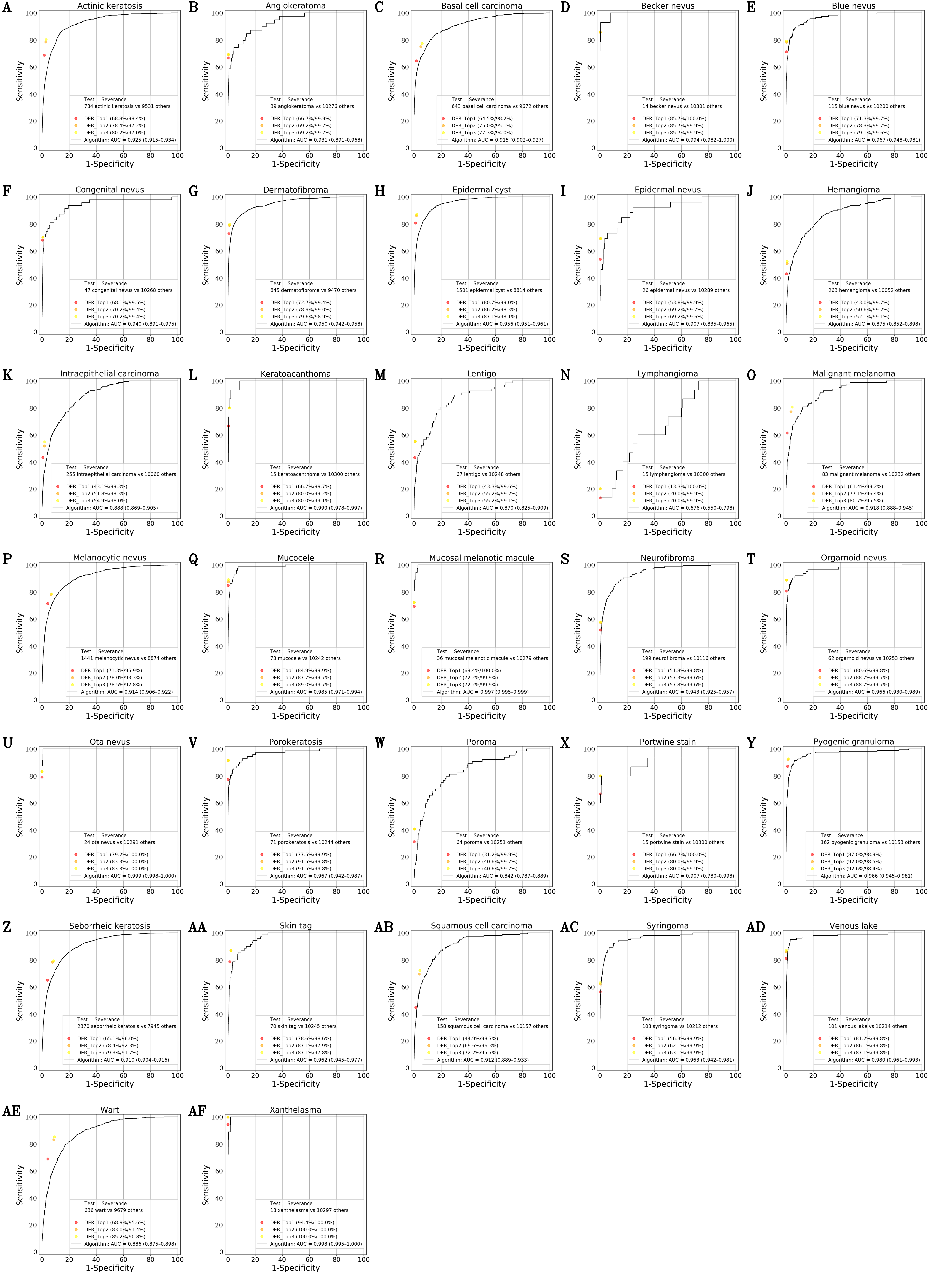

Supplement: S1 Fig — DER_Top1 –Top-1 accuracy of the clinical diagnoses. DER_Top2 –Top-2 accuracy of the clinical diagnoses. DER_Top3 –Top-3 accuracy of the clinical diagnoses. The algorithm analyzed multiple cropped images from the Severance Dataset B (39,721 images of 10,315 cases; 32 disorders). Not 32 outputs, but all 178 outputs were used for analysis without restriction. (TIF) [file pmed.1003381.s003.tif]

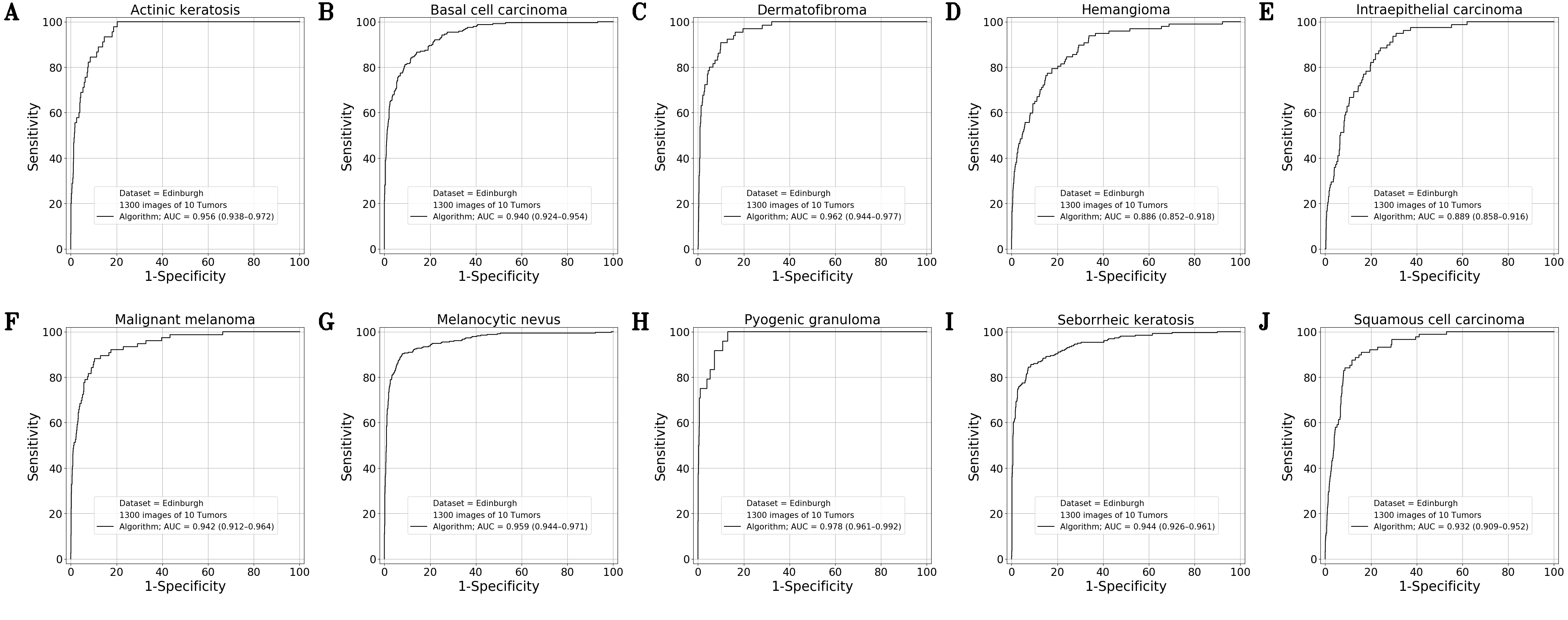

Supplement: S2 Fig — The algorithm analyzed 1,300 images from the Edinburgh dataset (https://licensing.edinburgh-innovations.ed.ac.uk/i/software/dermofit-image-library.html). All images in the Edinburgh dataset were cropped around the lesion of interest. All 178 outputs were used for analysis without restriction. We drew the ROC curves in a one-versus-rest manner. (TIF) [file pmed.1003381.s004.tif]
