# Supplementary material for: Assessment of deep neural networks for the diagnosis of benign and malignant skin neoplasms in comparison with dermatologists: A retrospective validation study
Source: PLoS Med. 2020 Nov 25;17(11):e1003381. doi: 10.1371/journal.pmed.1003381 (PMC7688128; doi:10.1371/journal.pmed.1003381)
Supplement: S1 Table — The algorithm analyzed multiple cropped images from Severance Dataset B (39,721 images of 10,315 cases; 32 disorders). We calculated the AUC values of the ROC curves in a one-versus-rest manner. (DOCX) [file pmed.1003381.s007.docx]

**S1 Table. Multiclass Task – AUCs and Top accuracies of the algorithm compared with those of clinical diagnoses for the 32 skin tumors of the Severance B Dataset**

|  |  |  | **Clinical Diagnosis** | | | **Algorithm** | | | |
| --- | --- | --- | --- | --- | --- | --- | --- | --- | --- |
| **No.** | **Class Name** | **Image Number** | **Top-1 Accuracy** | **Top-2 Accuracy** | **Top-3 Accuracy** | **Top-1 Accuracy** | **Top-2 Accuracy** | **Top-3 Accuracy** | **AUC (95% CI)** |
| 1 | Actinic keratosis | 784 | 68.8% | 78.4% | 80.2% | 26.4% | 42.1% | 52.3% | 0.925 (0.915–0.934) |
| 2 | Angiokeratoma | 39 | 66.7% | 69.2% | 69.2% | 46.2% | 56.4% | 64.1% | 0.931 (0.891–0.968) |
| 3 | Basal cell carcinoma | 643 | 64.5% | 75.0% | 77.3% | 47.0% | 59.7% | 66.6% | 0.915 (0.902–0.927) |
| 4 | Becker nevus | 14 | 85.7% | 85.7% | 85.7% | 42.9% | 42.9% | 50.0% | 0.994 (0.982–1.000) |
| 5 | Blue nevus | 115 | 71.3% | 78.3% | 79.1% | 71.3% | 82.6% | 84.3% | 0.967 (0.948–0.981) |
| 6 | Congenital nevus | 47 | 68.1% | 70.2% | 70.2% | 31.9% | 57.4% | 68.1% | 0.940 (0.891–0.975) |
| 7 | Dermatofibroma | 845 | 72.7% | 78.9% | 79.6% | 71.6% | 81.9% | 86.3% | 0.950 (0.942–0.958) |
| 8 | Epidermal cyst | 1501 | 80.7% | 86.2% | 87.1% | 56.0% | 70.2% | 77.5% | 0.956 (0.951–0.961) |
| 9 | Epidermal nevus | 26 | 53.8% | 69.2% | 69.2% | 26.9% | 26.9% | 30.8% | 0.907 (0.835–0.965) |
| 10 | Hemangioma | 263 | 43.0% | 50.6% | 52.1% | 30.8% | 46.8% | 57.4% | 0.875 (0.852–0.898) |
| 11 | Intraepithelial carcinoma | 255 | 43.1% | 51.8% | 54.9% | 16.5% | 28.6% | 34.5% | 0.888 (0.869–0.905) |
| 12 | Keratoacanthoma | 15 | 66.7% | 80.0% | 80.0% | 46.7% | 80.0% | 86.7% | 0.990 (0.978–0.997) |
| 13 | Lentigo | 67 | 43.3% | 55.2% | 55.2% | 35.8% | 47.8% | 53.7% | 0.870 (0.825–0.909) |
| 14 | Lymphangioma | 15 | 13.3% | 20.0% | 20.0% | 0.0% | 0.0% | 0.0% | 0.676 (0.550–0.798) |
| 15 | Malignant melanoma | 83 | 61.4% | 77.1% | 80.7% | 39.8% | 62.7% | 66.3% | 0.918 (0.888–0.945) |
| 16 | Melanocytic nevus | 1441 | 71.3% | 78.0% | 78.5% | 60.7% | 76.3% | 84.6% | 0.914 (0.906–0.922) |
| 17 | Mucocele | 73 | 84.9% | 87.7% | 89.0% | 27.4% | 45.2% | 47.9% | 0.985 (0.971–0.994) |
| 18 | Mucosal melanotic macule | 36 | 69.4% | 72.2% | 72.2% | 77.8% | 86.1% | 88.9% | 0.997 (0.995–0.999) |
| 19 | Neurofibroma | 199 | 51.8% | 57.3% | 57.8% | 43.7% | 57.3% | 62.3% | 0.943 (0.925–0.957) |
| 20 | Orgarnoid nevus | 62 | 80.6% | 88.7% | 88.7% | 62.9% | 79.0% | 82.3% | 0.966 (0.930–0.989) |
| 21 | Ota nevus | 24 | 79.2% | 83.3% | 83.3% | 54.2% | 66.7% | 75.0% | 0.999 (0.998–1.000) |
| 22 | Porokeratosis | 71 | 77.5% | 91.5% | 91.5% | 62.0% | 73.2% | 77.5% | 0.967 (0.942–0.987) |
| 23 | Poroma | 64 | 31.3% | 40.6% | 40.6% | 4.7% | 7.8% | 9.4% | 0.842 (0.787–0.889) |
| 24 | Portwine stain | 15 | 66.7% | 80.0% | 80.0% | 53.3% | 60.0% | 60.0% | 0.907 (0.780–0.998) |
| 25 | Pyogenic granuloma | 162 | 87.0% | 92.0% | 92.6% | 65.4% | 81.5% | 88.9% | 0.966 (0.945–0.981) |
| 26 | Seborrheic keratosis | 2370 | 65.1% | 78.4% | 79.3% | 48.5% | 64.6% | 72.2% | 0.910 (0.904–0.916) |
| 27 | Skin tag | 70 | 78.6% | 87.1% | 87.1% | 14.3% | 35.7% | 42.9% | 0.962 (0.945–0.977) |
| 28 | Squamous cell carcinoma | 158 | 44.9% | 69.6% | 72.2% | 28.5% | 47.5% | 56.3% | 0.912 (0.889–0.933) |
| 29 | Syringoma | 103 | 56.3% | 62.1% | 63.1% | 6.8% | 17.5% | 27.2% | 0.963 (0.942–0.981) |
| 30 | Venous lake | 101 | 81.2% | 86.1% | 87.1% | 40.6% | 55.4% | 65.3% | 0.980 (0.961–0.993) |
| 31 | Wart | 636 | 68.9% | 83.0% | 85.2% | 49.4% | 66.2% | 74.4% | 0.886 (0.875–0.898) |
| 32 | Xanthelasma | 18 | 94.4% | 100.0% | 100.0% | 72.2% | 88.9% | 88.9% | 0.998 (0.995–1.000) |
|  | *Mean ± STD* | 322 | 65.4±17.7% | 73.9±16.6% | 74.7±16.6% | 42.6±20.7% | 56.1±22.8% | 61.9±22.9% | 0.931±0.062 |

The algorithm analyzed multiple cropped images from Severance Dataset B (39,721 images of 10,315 cases; 32 disorders).

We calculated the AUC values of the ROC curves in a one-versus-rest manner.
