# Supplementary material for: Assessment of deep neural networks for the diagnosis of benign and malignant skin neoplasms in comparison with dermatologists: A retrospective validation study
Source: PLoS Med. 2020 Nov 25;17(11):e1003381. doi: 10.1371/journal.pmed.1003381 (PMC7688128; doi:10.1371/journal.pmed.1003381)
Supplement: S2 Table — The algorithm analyzed 1,300 images from the Edinburgh dataset (https://licensing.edinburgh-innovations.ed.ac.uk/i/software/dermofit-image-library.html). All images in the Edinburgh dataset were cropped images around the lesion of interest. We calculated the AUC values of the ROC curve in a one-versus-rest manner. (DOCX) [file pmed.1003381.s008.docx]

**S2 Table. Multiclass Task – AUCs and Top accuracies of the algorithm for 10 skin tumors in the Edinburgh Dataset**

| **No.** | **Class Name** | **Image Number** | **Algorithm** | | | |
| --- | --- | --- | --- | --- | --- | --- |
|  |  |  | **Top-1 Accuracy** | **Top-2 Accuracy** | **Top-3 Accuracy** | **AUC (95% CI)** |
| 1 | Actinic keratosis | 45 | 46.7% | 75.6% | 77.8% | 0.956 (0.938–0.972) |
| 2 | Basal cell carcinoma | 239 | 60.3% | 72.4% | 78.2% | 0.940 (0.923–0.955) |
| 3 | Intraepithelial carcinoma | 78 | 17.9% | 33.3% | 39.7% | 0.889 (0.858–0.915) |
| 4 | Dermatofibroma | 65 | 50.8% | 66.2% | 73.8% | 0.962 (0.945–0.978) |
| 5 | Hemangioma | 97 | 40.2% | 55.7% | 63.9% | 0.886 (0.850–0.917) |
| 6 | Malignant melanoma | 76 | 61.8% | 78.9% | 92.1% | 0.942 (0.914–0.965) |
| 7 | Melanocytic nevus | 331 | 79.8% | 90.3% | 94.0% | 0.959 (0.945–0.971) |
| 8 | Pyogenic granuloma | 24 | 62.5% | 79.2% | 87.5% | 0.978 (0.962–0.991) |
| 9 | Seborrheic keratosis | 257 | 68.5% | 80.5% | 87.2% | 0.944 (0.927–0.960) |
| 10 | Squamous cell carcinoma | 88 | 42.0% | 76.1% | 81.8% | 0.932 (0.909–0.951) |
|  | *Mean ± STD* | 130 | 53.0±17.4% | 70.8±16.1% | 77.6±16.1% | 0.939±0.030 |

The algorithm analyzed 1,300 images from the Edinburgh dataset (https://licensing.edinburgh-innovations.ed.ac.uk/i/software/dermofit-image-library.html).

All images in the Edinburgh dataset were cropped images around the lesion of interest.

We calculated the AUC values of the ROC curve in a one-versus-rest manner.
