# Supplementary material for: Assessment of deep neural networks for the diagnosis of benign and malignant skin neoplasms in comparison with dermatologists: A retrospective validation study
Source: PLoS Med. 2020 Nov 25;17(11):e1003381. doi: 10.1371/journal.pmed.1003381 (PMC7688128; doi:10.1371/journal.pmed.1003381)
Supplement: Attachment — Submitted filename: PMedicine_response_letter_R2.pdf [file pmed.1003381.s009.pdf]

Requests from the editors:

*We'd suggest revising the title to fit with PLOS Medicine's usual style as well as to ensure the importance and content of the article is clearer to a broad, multidisciplinary audience. Normally the title would begin with main concept/study question then the study design in the subtitle (ie, after a colon). eg, "Assessment of Deep Neural Networks for Skin Lesion Diagnosis in Comparison with Dermatologists: Retrospective Validation Study".

RESPONSE)
Thank you very much for your kind suggestion. We edited the title of our manuscript as follows:

“Assessment of Deep Neural Networks for the Diagnosis of Benign and Malignant Skin Neoplasms in Comparison with Dermatologists: Retrospective Validation Study”

*As noted by one reviewer, much of what is included in some of the figure legends probably belongs within the Methods section of the main text, and similarly some of the supplementary appendix may also need to be considered to move into the main text (and as main text figures) where that material is key to understanding the core methods and findings of the study.

RESPONSE)
Thank you for your insightful comment. We moved much of the key contents from the figure legends and the supplementary appendix to the main text in our revised manuscript.

LINES RELATED TO THIS ISSUE
Line 245~253, Line 321~328

*At this stage, we ask that you include a short, non-technical Author Summary of your research to make findings accessible to a wide audience that includes both scientists and non-scientists. The Author Summary should immediately follow the Abstract in your revised manuscript. This text is subject to editorial change and should be distinct from the scientific abstract. Please see our author guidelines for more information:
https://journals.plos.org/plosmedicine/s/revising-your-manuscript#loc-author-summary

RESPONSE)
Thank you for your comment. We included an Author Summary to our revised manuscript.

*Did your study have a prospective protocol or analysis plan? Please state this (either way) early in the Methods section.

a) If a prospective analysis plan (from your funding proposal, IRB or other ethics committee submission, study protocol, or other planning document written before analyzing the data) was used in designing the study, please include the relevant prospectively written document with your revised manuscript as a Supporting Information file to be published alongside your study, and cite it in the Methods section. A legend for this file should be included at the end of your manuscript.

b) If no such document exists, please make sure that the Methods section transparently describes when analyses were planned, and when/why any data-driven changes to analyses took place.

c) In either case, changes in the analysis-- including those made in response to peer review comments-- should be identified as such in the Methods section of the paper, with rationale.

RESPONSE)
This study was designed as a retrospective investigation, which made use of previously collected clinical photographs and medical records in order to test the diagnostic algorithm. We included all relevant IRB documents from multiple centers from which the images and medical records were collected.
Also, information on when this study was designed and when relevant data were collected has been included in the Methods section of our manuscript (January 1, 2008 – March 31, 2019).

LINES RELATED TO THIS ISSUE
Line 150~153

*In places, the English writeup for the paper could be improved (as there are a few non-grammatical phrasings) and therefore we'd suggest asking a native English speaking colleague or contact, if you can, to help proofread the revised version before resubmitting it. As examples, the following:
*Introduction line 83: "Imaging diagnoses using deep learning algorithm" - "Imaging diagnoses using deep learning algorithmS..."
*Introduction line 92: "However, clinical problems are more complex and relevancy of a single domain data employed for deep learning can quite vary.." >this does not flow/make sense very well and could be clarified. How about "However, clinical problems are more complex, and THE RELEVANCE of SINGLE DATA DOMAINS employed for deep learning can vary SUBSTANTIALLY"
*Introduction line 95: "images of the skin contain important relevant information, the amount of their importance is yet unknown in skin cancer diagnosis" > "images of the skin contain important AND relevant information, BUT the EXTENT OF THEIR importance is THUS FAR unknown FOR skin cancer diagnosis"
(These are just some quick examples, there may be more throughout the text so we'd recommend getting further input before resubmitting).

RESPONSE)
We appreciate your valuable comment. Our revised manuscript has been proofread by a native English speaking colleague before its resubmission.

Comments from the reviewers:

Reviewer #1: The authors provide an important report that sheds light on the current state of image based diagnostic AI for skin cancer, highlighting the limitations of reader studies that omit clinical metadata. Use of an external dataset for validation is a significant strength of the study.

The definition of 'malignancy output' is intriguing, and deserves additional explanation, perhaps in a supplementary table that specifically address the reasoning for inclusion of actinic keratoses (with a 0.2x modifier) and the inclusion of 'ulcer' as malignant.

RESPONSE>
Thank you very much for this comment. We included a detailed explanation on the malignancy output in the Supplementary Method section.
Chronic ulcers are uncommon and are a major risk factor of SCC development. On the other hand, acute ulcers are relatively common and not directly related to skin cancer. Unfortunately, image analysis cannot precisely distinguish chronic ulcers from acute ulcers. We decided to include ulcers in calculating the malignancy output as a premalignant condition, considering that the algorithm (which is designed as a screening tool) should warn the end user about the possibility of ulcers transforming into carcinomas. Similar reasoning can be applied to dysplastic nevi in Caucasians, but we did not include dysplastic nevi in the formula given the rare incidence of malignant melanoma in Asians. We believe this has to be validated in Caucasians in the near future.

The specific responses to this query are as follows:

1) The reason we chose 0.2× premalignant

Premalignant lesions such as actinic keratoses (AK) need particular attention and close observation. Therefore, lesions highly suspicious of AK (where AK output equals 1.0) were taken into account and were weighted as 0.2 because they would be otherwise neglected for the end user.

2) Why we chose 0.2 to weight premalignant lesions

Like physicians evaluated each lesion as "biopsy or not" / "malignancy or not", the algorithm generated reports in two different values, namely high-sensitivity threshold (formerly T90) and high-specificity threshold (formerly T80). The threshold for T80 and T90 without adding "0.2 × premalignant" were 0.2278 and 0.4451, respectively. We chose 0.2, which is close to the threshold of T80 (=0.2278) for lesions very much likely to be premalignant. For instance, lesions suspicious of AK with 100% certainty are given a weighted factor of 0.2, although it is not reported as being malignant ($0.2 < 0.2278$).

The final threshold for T80 and T90 when a weighted calculation of 0.2 × premalignant is performed turned out to be 0.2545 (=high-sensitivity threshold in this paper) and 0.4687 (=high-specificity threshold in this paper), respectively.

3) In fact, adding 0.2 × premalignant did not have a significant impact on AI's performance when tested with 2,844 images (PS + DER datasets of the previous paper).

AUC : 0.908 (no weight) vs 0.910 (0.2 × premalignant)

Average Precision for malignancy class : 0.637 (no weight) vs 0.632 (0.2 × premalignant)

Average Precision for benign class : 0.992 (no weight) vs 0.992 (0.2 × premalignant)

LINES RELATED TO THIS ISSUE
Supplement, Line 69~91

On a technical level: Soft-max outputs are not themselves designed to be subjected to thresholded analysis. They are relative scores, in relation to other outputs. Was the softmax layer removed before thresholding? This could significantly change results.

RESPONSE>
We obtained outputs ranging from 0 to 1 using the softmax layer as it is generally performed. Then, we applied thresholding. Just like you mentioned, the output from the softmax is a relative score, which has been standardized to a value between 0 and 1 after competition with other classes' outputs. The 178 classes comprise benign tumors, malignant tumors, various inflammatory disorders and normal skin. The final output is thus produced after competition with these various classes. Considering that malignant lesions tend to share bizarre morphology, we calculated the malignancy output in order to retrieve the common features of malignant lesions.

Authors should also explain why did they choose to study multiclass for only cropped images? Would suggest inclusion of multiclass performance on both cropped and non-cropped images, as well as a malignancy score.

RESPONSE>
In non-cropped (=unprocessed) images, there are usually many different lesions. For instance, if a patient visited the clinic with a mass suspicious of malignant melanoma, there might be other skin lesions such as nevi and seborrheic keratoses around that particular lesion suspicious of melanoma.

(A) Because the biopsy results served as the ground truth in this study, other surrounding lesions that were unintentionally photographed but not biopsied had to be excluded for analysis in the multi-class test. As shown in this study where the Top-1 accuracy of the attending physician reached only 65.4%, clinical impression based solely upon how the lesion looks like cannot be regarded as the ground truth.

(B) Even if we were to include the surrounding non-biopsied lesions, there should always be the question of whether we should include all of the tiny, inconspicuous lesions. In this situation, it is quite difficult to design inclusion criteria.

For the above mentioned reasons (A, B), we could not validate our multi-class classification with unprocessed images. Only lesions whose biopsy results were available were included in the validation of multi-class classification.

However, in the binary classification (cancer or not), we analyzed all surrounding lesions with the assumption that the ground truth of these surrounding lesions are all benign.

Lastly, more details might be included in the paper that highlight the quantifiable differences between the training and the test datasets.

RESPONSE

In this study, we aimed to validate the previously designed model (which was trained using different datasets) using the current test dataset.

We added explanations and comments regarding these datasets in the supplement method.

LINES RELATED TO THIS ISSUE

Supplement, Line 66~68

Reviewer #2: "Retrospective Assessment of Deep Neural Networks for 43 Skin Tumor Diagnosis in Comparison with Dermatologists in Real Practice" validates a previously-described machine learning algorithm (cited as [18], published as "Keratinocytic Skin Cancer Detection on the Face Using Region-Based Convolutional Neural Network", JAMA Dermatology, 2019) by mostly the same authors, on validation data obtained from Korea's Severance Hospital from 2008 to 2019. As discussed in the paper, this serves to reduce certain biases (e.g. selection bias, data leakage, generalizability, preselection) in evaluating the real-world performance of the algorithm. Additional supplementary performance on a publicly-available Edinburgh dataset of Caucasian subjects was also reported.

While dermatologist-level classification of skin lesions has been claimed since 2017 [citation 6], it remains true that many practical limitations and biases (some covered by the authors in the discussion) have complicated translation to actual practices. The particular strengths of this study lie in the retrospective validation on a dataset that seeks to represent the tumor distribution in an actual hospital. The main findings that automated diagnosis based on images only might not match up to standard clinical diagnosis, could be considered as valuable insight into the complications encountered in translating research systems to practice. That said, there may be a number of issues with the manuscript that warrant attention:

1. In the selection of the main study population (Severance Datasets), it is stated that "Only cases with a single lesion were included because of possible mismatch between the lesion and diagnosis in patients with multiple lesions" (Line 116). However, this would appear to undercut one of the main functionalities of the algorithm being validated, which is described in [18] as being able to detect multiple candidate lesions from unprocessed clinical images via R-CNN. Moreover, this further suggests that the study population might not be as unbiased as later promoted, since it has been restricted to patients with only a single lesion, beforehand.

RESPONSE>
We would like to thank you for your valuable comments.
“Patients with only a single lesion” actually means “patients with a single ‘biopsied’ lesions”. Thus, photographs of biopsied lesions also contain many other different skin lesions that are located in proximity. In the binary classification using unprocessed images, lesions around the biopsied one as well as those located in other parts (photographed altogether for the sake of keeping records) were also analyzed in this study, as shown in Figure 2. The algorithm suggested 13.4±16.9 (mean/STD) lesional boxes per one image file, and analyzed all of the possible lesions.

There are a few problems with cases where 2 or more biopsies were done:

(A) There were cases where multiple biopsies were done but there were only close-up photographs, which made it impossible to find the location of the lesions. There may be possible mismatch between the lesion and diagnosis in cases with multiple biopsied lesions.

(B) If there are two biopsied lesions in a single image, the cropped image analysis should be performed two times for the each crops, whereas in the unprocessed analysis, only one analysis is performed. This can be problematic in comparing the binary performance with unprocess images versus cropped images.

In particular, was this filtering of patients with multiple lesions, done before the initial step of the selection flowchart (Fig 1), since "multiple lesions" does not appear to be a listed exclusion step? The authors might disclose the number of patients with multiple lesions if so, and if possible explain further why testing on multiple lesions was not plausible (e.g. no lesion-level annotations?)

RESPONSE>
Thank you for your comment. As you pointed out, we revised Figure 1 to include the number of cases after exclusion of multiple biopsied cases.

The number of patients (patient N) should be distinguished from the number of cases (case N) because some patients may have had additional biopsies on different dates. Taking this into account, we revised Table 1 and related text in the main document.
Severance A : image N = 40331, patient N = 9556, case N = 10426
Severance B : image N = 39721, patient N = 9464, case N = 10315
Severance C : image N = 5065, patient N = 1304, case N = 1320

Because the pathologic diagnosis (obtained through biopsy) served as the ground truth in this study, only the 'main' lesions that were biopsied were used for the test of multi-class classification. For the binary classification, the final output was obtained for all lesions present in the images after testing using R-CNN under the assumption that the surrounding trivial lesions are all benign.

There exist problems in lesion-level annotation of the test dataset. First, defining the 'lesions' that need to be annotated is difficult. Close-up images of seemingly normal and healthy skin reveal some tiny red or brown spots or skin-colored papules. Therefore, to set up a reasonable criteria for defining 'lesions' is quite a challenge. Second, the surrounding non-biopsied lesions could not be tested because there was no information on their ground truth (i.e. pathologic diagnosis). Second, though clinical impressions of dermatology specialists could be used, they are always subject to error. Hence, we believe the lesion-level validation should be done exclusively for biopsied lesions, whose ground truths are available.

2. It is stated that "...normal images taken for record were included, even if they did not include the lesion of interest". Does this refer to misclassified diagnoses (i.e. some lesion is listed in the records, but the corresponding image does not show that lesion), or is there some other interpretation? In either case, the number of such cases might also be listed if possible.

RESPONSE>
For a patient with a BCC on the right cheek, not only the right cheek but also the left cheek is routinely photographed. The statement "...normal images taken for record were included, even if they did not include the lesion of interest" means that photographs of other areas (in this example, photographs of the left cheek) were also included for the analysis.

Related text has been edited based on Severance Dataset A (the legend of Figure 1 of the previous version referred to Severance Dataset B) Among 40,331 images of the Severance Dataset A, a total of 35,170 images contained lesions of interest, while the remaining 5,161 images were photographs without the lesions of interest.

LINES RELATED TO THIS ISSUE
Line 166~169

3. In general, there do not appear to be any representative validation dataset lesion images for either the unprocessed or the cropped analyses, unlike in say [18] and many other previous papers. The authors might consider including some such example images.

RESPONSE>
Thank you for your comment. We included an example image in our revised manuscript (Figure 2).

4. For the binary classification to determine malignancy experiment, it is stated that the "receiver operating characteristic (ROC) curves were drawn using the final output scores (the highest malignancy output in the unprocessed-image analysis and the average malignancy output in the cropped-image analysis)" (Line 156). However, it is not clear how these scores (from the unprocessed and cropped analyses) were combined. This might be clarified.

RESPONSE>
We included an example image (Figure 2) and comments in order to clarify this point.

One probability score is needed in order to draw the ROC curve, which in our study was the malignancy output. ROC curves were drawn using the threshold method. With a small increment of threshold value from 0 to 1, outputs greater than the threshold were reported as positive predictions.

In unprocessed-image analysis, the highest malignancy output was used as the final value because it analyses all skin lesions in the image, necessitating warning if there is any lesion suspicious of skin cancer. On the other hand, cropped-image analysis deals with a single skin lesion. Consequently, the average malignancy output was used as the final value.

Unprocessed-image analysis is a fully automatic process and can be used for screening purposes. On the other hand, cropped-image analysis cannot be fully automated because it requires the user to select the lesion of interest. Nonetheless, as shown in Figure S3, cropped-image analysis demonstrated slightly superior performance compared with the unprocessed-image analysis. (AUC 0.881 > 0.870; Delong's test, p=0.0022)

The predefined high-sensitivity and high-specificity thresholds are values that apply to the unprocessed-image analysis. They can also be applied to cropped-image analysis, a subject that we did not address in this study. (The real-time DEMO version of the cropped-image analysis is accessible on http://modelderm.com. It is performing binary decision using other datasets https://www.sciencedirect.com/science/article/abs/pii/S0022202X20301366)

LINES RELATED TO THIS ISSUE

Line 238~242, Figure 2 legend (line 535~539, line 542~544)

5. The derivation of malignancy output (Line 131) might be discussed. In particular, how were the weights defined (0.2 for actinic keratosis/ulcer, 1.0 for other outputs)? Was there some objective function or impact considered in the formulation of this equation?

RESPONSE>

In our revised manuscript, we included explanations on the formula used to calculate the malignancy output.

The specific responses to this query are as follows:

1) The reason we chose 0.2× premalignant

Premalignant lesions such as actinic keratoses (AK) need particular attention and close observation. Therefore, lesions highly suspicious of AK (where AK output equals 1.0) were taken into account and were weighted as 0.2 because they would be otherwise neglected for the end user.

2) Why we chose 0.2 to weight premalignant lesions

Like physicians evaluated each lesion as “biopsy or not” / “malignancy or not”, the algorithm generated reports in two different values, namely high-sensitivity threshold (formerly T90) and high-specificity threshold (formerly T80). The threshold for T80 and T90 without adding “0.2 × premalignant” were 0.2278 and 0.4451, respectively. We chose 0.2, which is close to the threshold of T80 (=0.2278) for lesions very much likely to be premalignant.

For instance, lesions suspicious of AK with 100% certainty are given a weighted factor of 0.2, although it is not reported as being malignant (0.2 < 0.2278).

The final threshold for T80 and T90 when a weighted calculation of 0.2 × premalignant is performed turned out to be 0.2545 (=high-sensitivity threshold in this paper) and 0.4687 (=high-specificity threshold in this paper), respectively.

3) In fact, adding 0.2 × premalignant did not have a significant impact on AI’s performance when tested with 2,844 images (PS + DER datasets of the previous paper).

AUC : 0.908 (no weight) vs 0.910 (0.2 × premalignant)

Average Precision for malignancy class : 0.637 (no weight) vs 0.632 (0.2 × premalignant)

Average Precision for benign class : 0.992 (no weight) vs 0.992 (0.2 × premalignant)

LINES RELATED TO THIS ISSUE

Supplement Line 69~91

6. The clinical diagnoses of the 65 attending physicians used for the binary classification experiment, might be further described. In particular, what protocol/clinical data was used/available? What is the experience level of the physicians if available (as stated for the readers), and were the images graded by multiple physicians with arbitration, or by single physicians only?

RESPONSE>
Thank you for this query. The clinical diagnosis is the most likely diagnosis given by the attending dermatology who actually saw the patient and ordered the biopsy. This clinical diagnosis was therefore the impression of one dermatologist recorded on the patient's medical record.
The vast majority of the attending dermatologists were board-certified dermatologists, with the exclusion of some 3rd, 4th year dermatology residents. There were mostly faculty members, but some clinical fellows were included too. They had an average of 7.1±9.5 (mean±SD) years of experience post-board certification at the time of their actual consultation and biopsy.

LINES RELATED TO THIS ISSUE
Line 155~158

7. In Line 177, the Fig 1 label does not appear to reflect the Top-1 clinical diagnoses discussed.

RESPONSE)
We apologize for this error. The sentence was actually referring to Figure 3 of the revised manuscript. Correction has been made accordingly in our revised version.

8. For binary classification of malignancy vs. benign, other than Top-1 and Top-3, a natural measure might be to aggregate the predicted probability of all malignant classes, against the predicted probability of all benign classes, in diagnosing malignancy. Additionally, it is not clear how binary classification would involve Top-k metrics, since the objective would appear to be to diagnose malignancy vs. benign, and not the specific tumor type.

RESPONSE>
We appreciate your comment.
Clinicians can provide Top1, Top2, Top3 diagnoses but not the probability score. In contrast, the algorithm can provide Top1, Top2, Top3 diagnoses along with the probability of each diagnosis. In addition, the malignancy output of the algorithm can be calculated with those probabilities.

For the binary classification (cancer or not), a ROC curve of the algorithm can be obtained because the algorithm gives probability scores (malignancy output). But for the clinicians, their results can only be drawn with three 'dots' because no probability score.

The specific clinical diagnosis can be transformed to binary decision (cancer or not). For example, if the following diagnoses were given from clinicians,

First impression of physician = Seborrheic keratosis
Second impression of physician = Basal cell carcinoma
Third impression of physician = Actinic keratosis

Then, because the Top-n accuracy is defined as the probability of the true diagnosis (= malignancy) being one of the (n) number of responses, each response would correspond to the following in the question of 'malignancy or not'

Top-1 = Benign
Top-2 = Malignancy
Top-3 = Malignancy

LINES RELATED TO THIS ISSUE
Line 216~227

Moreover, the relevant of a Top-k metric towards actual practical use is not very clear. For instance, given a malignant tumor, if the algorithm predicts a benign tumor as most probable, and the correct malignant tumor as second most probable, this is a true positive under Top-2; however, would this be referred by the algorithm for further attention? Conversely, if the algorithm predicts incorrect malignant tumor types for the three most probable diagnoses, this is a false negative, but the tumor would correctly be referred.

RESPONSE>
The algorithm not only gives its Top-1,2,3 predictions (diagnoses) but also reports specific output values (ranging from 0.0 to 1.0) for each prediction. For instance, please consider the following three different cases:

(A) Top-1 = SebK 0.33, Top-2 = Nevus 0.32, Top-3 = Melanoma 0.31
(B) Top-1 = SebK 0.33, Top-2 = Melanoma 0.32, Top-3 = Nevus 0.31
(C) Top-1 = SebK 0.97, Top-2 = Melanoma 0.02, Top-3 = Nevus 0.01

In case (A), both Top-1 and Top-2 predictions are benign conditions. But the Top-3 prediction, which is malignant melanoma, cannot be neglected because its output value is relatively high (0.31). In case (B), Top-2 and Top-3 predictions have been switched in order, but essentially all three possible predictions have to be considered. In case (C), the Top predictions are the same as those of the case (B). However, the output values of Top-2 and Top-3 predictions are very low, meaning that the lesion of interest is a seborrheic keratosis with a very high level of certainty.

In order to generate a reliable metric that can reflect the possibility of a lesion being a malignancy, we calculated the malignancy output. The formula comprises both malignant and premalignant conditions among all output values (from Top-1 to Top-178). In case (C), the malignancy output is quite low, whereas in cases (A) and (B), the algorithm reports a relatively high malignancy output although the Top-1 prediction is a benign condition.

To maximally benefit from the algorithm, potential users should have a fairly good understanding of what Top-1,2,3 outputs as well as the malignancy output mean.

9. For the presented main results (Figure 2), how was the gold standard diagnosis (against which both the algorithm and the physicians/readers were compared against) obtained?

RESPONSE>
Since all of the 10,426 cases included in this study were those with diagnoses confirmed by pathologic examination, the pathologic diagnosis served as the gold standard diagnosis (ground truth). "Malignancy" in Table 1 was defined as being one of the following malignant conditions: angiosarcoma, BCC, DFSP, SCC in situ, Kaposi sarcoma, keratoacanthoma, melanoma, Merkel cell carcinoma, and SCC.

LINES RELATED TO THIS ISSUE
Table 1, Line 153~155

10. For the accuracy against number of algorithm's used outputs graphs (Figure 3), the set of number of outputs for which actual performance was obtained might be provided (i.e. was every number between 32 and 178 tested, and if not, which numbers were tested and the remainder interpolated?)

RESPONSE>
The test datasets included only 32 possible diagnoses, whereas the algorithm considers a total of 178 diagnoses. The performance can be optimized by ruling out all other outputs (by making them '0') if the correct diagnosis exists among the 32 possible outputs. A number of possible diagnoses raging from 32 to 178 were selected, and the ones that were not selected were assigned to an output value of '0'.

32 selected dx = 32 dx
33 selected dx = 32 dx + randomly selected 1 dx among 146 dx
34 selected dx = 32 dx + randomly selected 2 dx among 146 dx
…
…
176 selected dx = 32 dx + randomly selected 144 dx among 146 dx
177 selected dx = 32 dx + randomly selected 145 dx among 146 dx
178 selected dx = 178 dx

Because random selection generates a huge number of possible combinations, this process was repeated 2,000 times before getting a final result (bootstrap method of statistics). Every number between 32 and 178 was tested.

In actual clinical settings, the problem is not always a multiple choice question. Hundreds of different diagnoses should be considered before making a clinical diagnosis. Various conditions including nummular eczema, prurigo nodularis, and keloid can mimic cutaneous neoplasms. Therefore, a large number of possible diagnoses (certainly over 178) should be considered in order to get to the correct diagnosis.

The result of Figure 4 is why the algorithm, which outperforms us in well-defined, testing circumstances, may not perform as outstandingly in real life settings. This explains the 'hype' that has been questioned in this field. The algorithm shows incredible performance in a controlled

environment, but is less promising in solving un-trained problems or problems where there are numerous possible answers.

LINES RELATED TO THIS ISSUE
Line 322~328

11. While the algorithm being validated has been described in a previous publication [18], the authors might consider more fully covering its details (e.g. training parameters, how the various component CNNs were combined) in the supplementary appendix.

RESPONSE>
Thank you for this valuable point. In our revised manuscript, details on the training process were added. The results of the training do not largely depend on the hyperparameters or which CNN used. We wanted to emphasize that the analysis of our model consisted of three distinct stages, which we included in the revised version of our manuscript.

LINES RELATED TO THIS ISSUE
Supplement Line 27~64

12. Much of the information presented as Figure descriptions/captions might be more appropriately located in the main text.

RESPONSE>
Thank you for your insightful comment. We moved much of the key contents from the figure legends and the supplementary appendix to the main text in our revised manuscript.

LINES RELATED TO THIS ISSUE
Line 166~169, Line 245~253, Line 322~328

13. A number of minor grammatical/spelling issues might be addressed, e.g. "were higher 88.1% / 83.8% of" (Line 176), "hospital achieves usually include" (Line 231), "skin cancer diagnosis is proved to be a difficult task" (Line 274); overall, the authors might consider having the manuscript proofread further.

RESPONSE>
We apologize for this issue. The revised version of our manuscript has been proofread and corrected for grammatical and spelling errors.

Reviewer #3: The authors validate a CNN for the diagnosis of skin tumors in comparison with dermatologists on an external validation set of 40,331 images. It is a large study that has merit and depth but needs some improvement.
1. The expression "skin tumor" in the title and in the main text of the manuscript is misleading because it suggests that the target diseases are only malignant neoplasms. In colloquial language skin tumor means skin cancer. The title suggests that the test set included 43 types of skin cancer. The categories included in the test set include benign neoplasm, malignant neoplasms, hamartomas, and cysts. Lentigo, for example, is not a tumor, it is flat. Cysts are not tumors. The generic term tumor should be avoided. The title should be something like "43 classes of solitary skin lesions" or "43 classes (categories) of common, non-inflammatory solitary skin lesions", but certainly not skin tumors.

RESPONSE>
Thank you very much for your valuable comments. Considering the suggestions from the Editors, we edited the title of our manuscript as the following:

"Assessment of Deep Neural Networks for the Diagnosis of Benign and Malignant Skin Neoplasms in Comparison with Dermatologists: Retrospective Validation Study"

2. Data availability (training set, test set, and code) is very restrictive and is probably not in line with the open data policy of the PLOS journal family. Authors should rethink their decision. Also the code of the CNN should be made available to let others repeat the experiments.

RESPONSE>
Thank you for your query. This study was designed as a validation study using an algorithm that has been trained previously. Details on the training have already been published in our previous articles and a real-time web Demo has been made accessible to the public since last year, to demonstrate reproducibility of our results.

The region-based CNN(Faster-RCNN) and CNN(SENet) used in this study were published in engineering journals by Shaoqing Ren and Jie Hu, respectively, and the training and test codes are all publicly available. The codes of faster-RCNN is available at https://github.com/rbgirshick/py-faster-rcnn, and those of of the SENet is also available at https://github.com/hujie-frank/SENet, and there are several tutorials for fine-tuning of imagenet pretrained models (for example,cat vs dog; http://adilmoujahid.com/posts/2016/06/introduction-deep-learning-python-caffe/).

The performance of our model does not depend much on the choice of CNN model or hyperparameters, but is greatly affected by the dataset. Considering the large number of clinical images that were collected in this study, we are afraid that making all original clinical images available for the public may violate the regulations from the IRB.

To demonstrate reproducibility, we released a real-time web Demo (http://rcnn.modelderm.com for the unprocessed-image analysis; http://modelderm.com for the cropped-image analysis). The performance of the model can be tested more accurately by actually using it.

We offer thumbnail images of all patients from the Severance dataset (https://figshare.com/articles/The_Severance_Validation_Data_set/11058764). This will provide the characteristics and distribution of diseases. The demographic information (age/sex) and biopsied sites were also provided with an Excel file.

3. The results section needs some revision. Outcome measures with confidence intervals should be reported instead of p-values only.

RESPONSE>
Thank you for your suggestion. We reported the mean values with confidence intervals throughout the Results section.

4. In the results section the authors wrote: As shown in Fig 2B, overall, the sensitivities and specificities of the algorithm were comparable with those of the participants (Wilcoxon test; P=0.99/0.0043 for sensitivity/specificity of the Top-3 of the participants versus sensitivity/specificity of the algorithm at the high-sensitivity threshold; P=0.61/0.097 for sensitivity/specificity of the Top-1 of the participants versus sensitivity/specificity of the 196 algorithm at the high-specificity threshold). It is very difficult to understand this sentence and to extract the correct numbers for the outcome measures sensitivity and specificity. It is inappropriate to give only P-values without outcome measures. An easier metric would be to report the number of correct diagnosis by batch.

RESPONSE>

First, we would like to first apologize for the confusion. The issues you mentioned regarding the statistical analysis are addressed point by point in the revised manuscript (Line 300~312).

There are two ways to evaluate the diagnostic accuracy: (1) "correct exact diagnosis", (2) "correct binary decision"

(1) Evaluation of the diagnostic accuracy using "the number of correct exact diagnosis by batch" corresponds to the Top-1 accuracy comparison in multi-class classification. In that sense, we observed significant difference among the diagnostic accuracy of the algorithm, reader test participants and the actual consulting dermatologists:

Clinical Diagnosis (68.1%, CI 65.2–71.0) > AI (49.5%, CI 46.7–52.4) > Reader Test Participants (37.6%, CI 34.4–40.9)

(2) Evaluation of the diagnostic accuracy using "the number of correct binary decision by batch" is more suitable to assess the ability to predict a cancerous condition because the previous method may correctly identify a malignancy but fail to report the exact type of tumor.

Prior to direct comparison, the clinical diagnosis recorded by the actual consulting dermatologist was converted to binary decision (Line 216~227). The sensitivity of malignancy detection is improved when when we look at all three clinical diagnoses and see whether at least one malignant condition is included. Conversely, if we consider only the Top-1 diagnosis and see whether it correctly diagnosed malignancy, the diagnostic specificity is improved. For these reasons, we separately compared the diagnosis of the algorithm with that of the clinicians in two different ways as described below:

algorithm (high-sensitivity threshold decision) <-> doctor (binary decision from Top-3)
algorithm (high-specificity threshold decision) <-> doctor (binary decision from Top-1)

LINES RELATED TO THIS ISSUE
Line 216~227

5. The authors further wrote: We calculated mean accuracy by averaging the accuracies of the 32 disorders as follows: macro-averaged mean Top-(n) Accuracy = (Top-(n) Accuracy of actinic keratosis + Top-(n) Accuracy of angiokeratoma + … + Top-(n) Accuracy of xanthelasma) / 32. This fits better to the methods section and is also difficult to understand. I am not sure but could it be that the authors refer to the average recall or the mean sensitivity? It needs clarification.

RESPONSE>
Thank you for this query.

The accuracy refers to the probability to draw correct answers to the given questions.

Accuracy = (True Positive + True Negative) / (All Cases)

On the other hand, recall (=sensitivity) is the probability of correctly predicting cancers among all cancer cases.

Recall = Sensitivity = (True Positive) / (True Positive + False Negative)

We added a detailed explanation on the accuracy, and relevant text has been moved to the Methods section as per your suggestion.

LINES RELATED TO THIS ISSUE
Line 245~253

6. It is also unclear if the ± refers to confidence intervals or standard deviations.

RESPONSE>
± denotes the standard deviation. We clarified this point in our revised manuscript.

7. When the authors speak of accuracy in the multiclass problem do they mean frequency of correct diagnoses? Please clarify

RESPONSE>

We appreciate your comment. The definition of accuracy is as follows:
Top-(n) accuracy is the percentage that a model renders a correct diagnosis within its (n)th choice.

Just like you mentioned, the accuracy of the multiclass problem was defined as the probability of getting the correct diagnosis.

LINES RELATED TO THIS ISSUE
Line 245~253

8. It is unclear how the 1,320 patients were selected from the master set to create the batches for the reader study.

RESPONSE>

The reader test was performed with 44 dermatologists. Each reader test consisted of 30 different cases. Thus, a total of 1,320 cases were selected from Severance B Dataset (10,315 cases) to test both binary and multi-class classification. Using the random function in Python, 1,320 different numbers between 1 and 10,315 were randomly selected.
Therefore, a total of 1,320 (44 x 30 = 1,320) cases were selected to create the batches for the reader test.

LINES RELATED TO THIS ISSUE
Line 230~232

9. Much more information is need from the 44 dermatologists who participated in the reader study and how they were selected. Is there a risk of self selection bias, recruitment bias etc.

RESPONSE>
The reader test was performed by 8 dermatologists from Severance Hospital and 36 dermatologists from other institutions. Because the test images were from the Severance dataset, senior staff who actually consulted the included patients did not participate in the reader test. Thus, from Severance Hospital, only junior staff (6 dermatologists with 1 year of experience after board certification, 1 dermatologist with 2 years of experience after board certification, and 1 dermatologist with 4 years of experience after board certification) took the reader test.

For all participants in the reader test, years of practice experience after board certification was an average of 5.7±5.2 (mean±SD) years. It was also found that the attending dermatologists in the Severance Hospital had 7.1±9.5 (mean±SD) years of practice experience after board certification at the time of actual consultation and biopsy, which does not differ greatly from the participants to the reader test.

LINES RELATED TO THIS ISSUE
Line 155~158, Line 232~236
