# Supplementary material for: Assessment of deep neural networks for the diagnosis of benign and malignant skin neoplasms in comparison with dermatologists: A retrospective validation study
Source: PLoS Med. 2020 Nov 25;17(11):e1003381. doi: 10.1371/journal.pmed.1003381 (PMC7688128; doi:10.1371/journal.pmed.1003381)
Supplement: Attachment — Submitted filename: Response Letter R3.pdf [file pmed.1003381.s010.pdf]

Requests from the editors:

Abstract

Please add further background here to provide context for this study and help readers understand the importance of this work

RESPONSE

Thank you very much for your review. We edited the abstract to improve reader understanding.

LINES RELATED TO THIS ISSUE

Line 47~50

Please add further information about the datasets used and the clinicians who participated. It would be helpful to distinguish between 65 attending physicians and 44 dermatologists to note their specific role in this study.

RESPONSE

We appreciate your comment. Additional information about the datasets and the clinicians mentioned in this study has been included in the revised abstract.

LINES RELATED TO THIS ISSUE

Dataset : Line 57~60

65 Physicians and 44 dermatologists : Line 62~63, Line 66~68

The last sentence of the methods and findings section must include 2-3 limitations of your work

RESPONSE

Thank you for this comments. Limitations of this study has been included in the methods and findings section.

LINES RELATED TO THIS ISSUE

Line 85~86

Conclusions

Please temper these, especially “a mass screening tool in a telemedicine setting” as this was not directly tested.

RESPONSE

We highly appreciate your comment. As per your suggestion, we deleted the phrase “a mass screening tool in a telemedicine setting” in the conclusions.

Please ensure that the study is reported according to the STARD 2015 reporting guideline for diagnostic accuracy studies, and include the completed STARD checklist as Supporting Information. Please add the following statement, or similar, to the Methods: "This study is reported as per the STARD 2015 reporting guideline for diagnostic accuracy studies (S1 Checklist)."

The STARD guideline can be found here: http://www.equator-network.org/reporting-guidelines/stard/

When completing the checklist, please use section and paragraph numbers, rather than page numbers.

RESPONSE

Thank you very much for this valuable query. The Main text as well as the abstract have been edited to meet the STARD guideline. We have completed the checklist, which we attached to the Supplement.
LINES RELATED TO THIS ISSUE
Line 168

Author Summary
Lines 86-95 should be simplified as it is meant to be a non technical summary. Please replace "convolution neural networks" and "lesions" with less technical language to convey that the aim of this work is to compare the diagnostic efficiency of an algorithm versus a dermatologist.

RESPONSE
Thank you for your comment. We edited the relevant text in our revised version with less technical language.
LINES RELATED TO THIS ISSUE
Line 98, 102~103, 120~121

Line 98- please avoid assertions of primacy
RESPONSE
We appreciate your comment. We omitted 'the first' in our revised Author Summary.

Line 113-quality of images is not mentioned previously in this section and I would suggest this is removed or further context provided in earlier bullet points
RESPONSE
Thank you for your kind comment. We edited the relevant text accordingly.

Introduction

Please add a space between text and reference brackets throughout the text. Please note all full stops should be after square brackets.
RESPONSE
We appreciate your comments. Our manuscript has been edited accordingly.

Please clearly state the aim of your work towards the end of the introduction.
RESPONSE
Thank you for this comment. In the revised version of our manuscript, the aim of our work has been clearly stated.
LINES RELATED TO THIS ISSUE
Line 161~164

Please also briefly provide context of the algorithm and any previous work describing it.
RESPONSE
Thank you for this query. The context of the algorithm and the results of previous work have been added in the revised manuscript.
LINES RELATED TO THIS ISSUE
Line 156~161

Please consider if this sentence is relevant "the complete sequence and location of white and black stones are required to play game Go; thus, DeepMind's AlphaGo[17] was trained with this data to surpass human champions". We recommend removing this.

RESPONSE
Two main characteristics of fields where deep learning shows outstanding performance (e.g. computer games, Go, chess) are : 1) that virtually all relevant data can be acquired along with all sequences, and 2) that all data can be quantified.
In contrast, it is impossible to gather all relevant data in the field of medicine, and the data are mostly qualitative. Therefore the data used for algorithm training is subject to limited relevancy. Nowadays people expect that the algorithm would always outperform human champions, as did Alphago. However, some regard this much of an expectation for the algorithm in the field of medicine as the 'hype'. In fact, the algorithm's superiority cannot be easily reproduced in medicine because, unlike in chess or Go, the algorithm can never be trained with all relevant information.

LINES RELATED TO THIS ISSUE
Line 140~141

Methods
Did your study have a prospective protocol or analysis plan? Please state this (either way) early in the Methods section. Please explicitly state that none of these analyses were pre-planned, as requested in the previous version.

RESPONSE
Thank you for this query.
This study was initially planned in 2019, and did not include any prospective analysis protocol or plan. The retrospective nature of this study is stated several times in the manuscript, including the abstract.
However, Department of Dermatology, Severance Hospital has long been operating a dedicated prospective protocol to manage all biopsy cases. Clinical diagnosis, pathologic diagnosis, lesion site, biopsy site, biopsy lesion, slide number, information of special stains for all biopsy specimens, and name of attending physicians have been kept and organized according to this protocol since 2005.

LINES RELATED TO THIS ISSUE
Line 169~177

Please provide additional information regarding where this study was carried out (city,country), where were the patient images obtained from and where were the dermatologists based.
Can you briefly provide the information about severance data sets A ,B,C in the main text?

RESPONSE
Thank you very much for this query.
This study was carried out in Seoul, Republic of Korea. All institutions from where the photographs were collected are located in Seoul. Also, all dermatologists who participated in the reader test were based in Seoul, and cities around Seoul.

As per your suggestion, we provided brief information on Severance sub-datasets in the main text, which was previously included in the figure legends.

LINES RELATED TO THIS ISSUE
Line 171, 290
Line 213~223

Results
Please provide exact p values where appropriate, except when p<0.001. Please also ensure all p values are provided up to three decimal places

RESPONSE
Thank you for your comment. all p values are provided up to three decimal places in our revised manuscript.

Discussion
Please present and organize the Discussion as follows: a short, clear summary of the article's findings; what the study adds to existing research and where and why the results may differ from previous research; strengths and limitations of the study; implications and next steps for research, clinical practice, and/or public policy; one-paragraph conclusion.

RESPONSE

We deeply appreciate your kind suggestion. As per your suggestion, the following changes have been made to our manuscript:
(1) a short, clear summary of the article's findings; Line 415~420
(2) what the study adds to existing research and where and why the results may differ from previous research; Line 421~525
(3) strengths and limitations of the study; Line 526~540
(4) implications and next steps for research, clinical practice, and/or public policy; Line 541~562
(5) one-paragraph conclusion; Line 565~573

Lines 351-353 require revision for clarity and to avoid the double use of “studies”

RESPONSE
We appreciate your valuable comment. The sentence has been edited in our revised manuscript to avoid ambiguity and redundant use of the word “studies”.

LINES RELATED TO THIS ISSUE
Line 428~430

Line 372 should be revised “ do not represent the general population”
RESPONSE
Thank you for this comment. The sentence has been revised accordingly.
LINES RELATED TO THIS ISSUE
Line 452

In the limitation section you mention an Edinburgh dataset which has not been mentioned before. Please provide the necessary details in the methods and results section

RESPONSE
Thank you very much for this comment.

To accommodate your request, we include a short summary of the Edinburgh dataset in the main text.

In our revised manuscript, we added the explanation on the above six different test configurations. Also, we have indicated that the detailed results for the Edinburgh dataset are shown in the Supplement section.

In this study, we performed multiple validations tests in six different configurations:

Main Text
(a) Binary classification with the unprocessed images of the Severance dataset A
(b) Binary classification with the cropped images of the Severance dataset B
(c) Binary classification with the unprocessed images of the Severance C – Reader test
(d) Binary classification with the cropped images of the Severance C – Reader test

Supplement
(e) Multiclass classification with the cropped images of the Severance dataset B
(f) Multiclass classification with the cropped images of the Edinburgh dataset

For those unfamiliar with the subject, the meaning of multiclass and binary classification may be somewhat confusing. Therefore, we are afraid that the results of multiclass classification in the Supplement may cause more confusion to the reader if included in the Main Text because the Main Text contains technical data processing such as the conversion of multiclass classification to binary decision.

Considering that the binary decision may be of primary interest for the reader and from the clinical perspective, it has been included in the Main Text. The multiclass classification has been separately described in the Supplement.

LINES RELATED TO THIS ISSUE
Line 294~305
Line 362~363

Please add citations to support this statement “as melanoma is relatively rare in 466 Asians”.

RESPONSE
Thank you very much for your comment. The following citations have been added to our revised manuscript:

https://pubmed.ncbi.nlm.nih.gov/28707459/
Korea (0.58 in men and 0.66 in women per 100 000 populations)

https://pubmed.ncbi.nlm.nih.gov/28609317/
Japan (in-situ = 0.41 and invasive = 1.24 per 100 000 populations)
Taiwan (0.65)
USA (21.8) , New Zealand (39.4 in men and 35.8 in women)

LINES RELATED TO THIS ISSUE
Line 536

Please do not use the term Caucasians and use “white” instead.
RESPONSE
We truly appreciate your suggestion. We replaced the term Caucasians with White throughout the entire manuscript.

Data statement- please can you clarify if the algorithm is available to interested researchers, providing a link for access whether free or requested here. For the test subset, please provide a contact person for data requests. Please note that in both instances, authors cannot act as sole sources of the data.

RESPONSE
We would like to thank you for this query.

Unlike X-ray or dermoscopy images, clinical photographs used in this study contain personally identifiable information (PII) which depict unique features of the patients’ intimate body areas and anatomical structure. This is why approval is required from the Institutional Review Board (IRB) to even perform internal testing with the clinical photographs.

Disclosure of these datasets to third parties requires, at the minimum, the approval of the IRB of Severance Hospital.

Even if the necessary approvals could unanimously be attained, we would have to address the legal and regulatory issues arising from the disclosure. Furthermore, there are likely to be broader societal and ethical ramifications, not least any public outcry from the stern advocates of right to privacy.

We would like to also point out that in review articles regarding the use of deep learning algorithm in dermatology (https://www.jmir.org/2018/10/e11936/, https://www.sciencedirect.com/science/article/abs/pii/S0022202X2031201X), only 'a few' datasets used in published papers are available to the public.

Although we are not able to accommodate your request for the datasets for the reasons above, we understand that the test dataset needs to be shared among investigators to allow comparison between studies. That is why we carried out additional validation using the Edinburgh dataset, which is accessible to all.

Regarding data availability, we have added in our manuscript that we may provide the model in the form of API (application programming interface) if a large set of data is necessary for additional validation.
However, we regret to say that disclosure of the model is not possible for the following reasons:

1) Possibility for release of 'copy models' after slight modification or fine-tuning of our model
2) Possible commercial use of our model without permission. We have not yet made the offline model available for the public because it portends a risk of unrestricted and unauthorized commercialization.

In contrast to models dealing with other medical data such as radiologic images that can only be employed by medical professionals, our model can easily be commercially released to the public in the form of smartphone applications, for example.

To the best of our knowledge, two of our papers published in medical journals (http://www.jidonline.org/article/S0022-202X(18)30111-8/fulltext , https://doi.org/10.1371/journal.pone.0191493) are the only ones where the model used in the study of clinical photographs was made available to the public. We believe that the models used in other papers (*nature*; https://www.nature.com/articles/nature21056, *nature medicine*; https://www.nature.com/articles/s41591-020-0842-3) were not made public for the same reasons mentioned above.
We do however understand that model disclosure is essential to demonstrate reproducibility of our results. Therefore, we have made a real-time web-demo available (http://modelderm.com , http://rcnn.modelderm.com) since 2018.
Most practicing dermatologists may not be familiar with direct model handling. Thus, we believe that a web-demo of our model is more suitable than releasing the model file itself. Whereas the model we posted online in 2017 (https://figshare.com/articles/software/Caffemodel_files_and_Python_Examples/5406223) was downloaded only 141 times, the web-demo is being actively tested (a daily average of 30 times).

There also have been academic letters discussing its performance (https://pubmed.ncbi.nlm.nih.gov/29864435/ , https://pubmed.ncbi.nlm.nih.gov/29864434/).

LINES RELATED TO THIS ISSUE
Line 592~593

Bibliography must be in Vancouver style
RESPONSE
Thank you for your comment. Bibliography has been edited according to the Journal's style in our revised version.

Most of the information in figure legends is not provided in the methods section. This was specifically requested in the previous version of the manuscript. Specifically, the information about various datasets has not been included in the methods section and how unprocessed image analysis versus cropped image analysis is carried out is also not in the methods.

RESPONSE
We deeply appreciate your comment. We added the information listed in the figure legends to the methods section of the main text.
LINES RELATED TO THIS ISSUE
Line 201~212
The information about various datasets - Line 213~223
How unprocessed image analysis versus cropped image analysis was carried out - Line 245~254

Is the information on page 27 lines 578 onwards intended to be a figure legend? I am not sure this is appropriate, please place this information in the results section. The same goes for text on Page 30 which should be in the methods section only. I note that you have added lines 166-169 but it needs to be in the same level of detail as page 30.

RESPONSE

Thank you for your valuable comment. The information on page 27 has been moved to the results and discussion sections. (Line 396~398 / Line 442~451)

The text on page 30 has been moved to the methods section. (Line 208~212)

Comments from the reviewers:

Reviewer #2: We thank the authors for addressing most of our concerns from the previous review round. A few points might be however further considered:

1. In the response to Point 1, it is stated that "photographs of biopsied lesions also contain many other different skin lesions that are located in proximity". In such cases, how is the actual targeted biopsied lesion identified (e.g. is its position specified in a separate data/annotation file, or is it assumed to be obvious from the given label)? This might be described in the text.

RESPONSE
In case there are multiple skin lesions nearby the one to be biopsied, that particular lesion to be biopsied was easily identified with the help of the following factors:

(1) Detailed description on the exact location (‘proximal phalanx of right index finger’ rather than ‘finger’) of the biopsied lesion was available in the ‘Biopsy Request Form’.
(2) Marking with a surgical marker or by placing a surgical ruler right next to the lesion to be biopsied.
(3) The biopsied lesion almost always located right in the center of the photograph
(4) Referring to the clinical diagnosis listed in the medical records to identify the biopsied lesion

Ambiguity arises when there are multiple similar lesions within a small boundary (for example, seborrheic keratoses, angiokeratomas, actinic keratoses and kaposi sarcomas), which makes it difficult to know the exact lesion that was biopsied.
The corresponding cases were found to be 2.46% (257 out of 10426 cases) of the total number of cases. Specifically, we identified 32 such cases within the malignancy group (top two diagnoses being kaposi sarcoma and bowen’s disease with 13 and 10 cases, respectively) and 225 cases within the benign group (top two diagnoses being seborrheic keratosis and wart with 70 and 44 cases, respectively). Here we provide the number of cases where the lesion biopsied could not be exactly identified together with the pathologic diagnosis:

actinic keratosis 17, angiokeratoma 6, basal cell carcinoma 4, intraepithelial carcinoma 10, dermatofibroma 18, epidermal cyst 1, hemangioma 19, melanocytic nevus 17, neurofibroma 9, porokeratosis 11, seborrheic keratosis 70, skin tag 1, squamous cell carcinoma 3, syringoma 12, wart 44 (flat wart = 26, common wart =18), angiosarcoma 2, kaposi sarcoma 13

The reason why we decided to include all similar lesions as one case :
(1) If we include only one lesion, only photographs of typical lesions would be selected, which may lead to selection bias
(2) Lesions including kaposi sarcomas, flat warts, and angiokeratomas naturally tend to present as multiple grouped lesions. Thus, the biopsy report should reflect the pathologic diagnosis for all similar grouped lesions.

LINES RELATED TO THIS ISSUE
Line 188~196

2. Reference is made to "inherently impossible" cases. Exactly why these cases are impossible might be briefly explained (e.g. label not present in training set, etc)

RESPONSE

Some cases were classified as 'inherently impossible' for either of the following reasons:

The exact location of the lesion was not clear. For instance, lesions such as morpheaform BCC and epidermal cyst are sometimes difficult to locate because they tend to blend into the background. Thus, if there was any uncertainty regarding the lesion's exact location within the photograph, we classified these cases as 'inherently impossible'. Epidermal cysts was the most common diagnosis for such cases (497 cases).

Therefore, 'inherently impossible' cases were the ones where we could not identify the lesion to be cropped. In contrast, photographs that were not adequate for analysis were classified as 'inadequate', separately from 'inherently impossible' cases.

The cases you mentioned where "*the labels were not present in the training set*" consist of a total of 5 classes: Spitz nevus, dermatofibrosarcoma protuberans, angiosarcoma, Kaposi's sarcoma, and Merkel cell carcinoma. They were included in the binary classification (to determine 'cancer or not'), whereas they were excluded from the multi-class classification because the algorithm was not trained with those classes.

LINES RELATED TO THIS ISSUE
Line 197~199

3. The response for Point 8 was helpful in understanding the details of the algorithm assessment, and the authors might consider including a possibly-abridged version in the supplementary material.

RESPONSE
We appreciate your insightful comment. We have added the response for Point 8 to the Supplement.

LINES RELATED TO THIS ISSUE
Supplement Line 95~117

4. In line 385/409, does "innocent bias" have some widely-accepted technical meaning? If not, it might be termed as "hindsight bias" or "leakage bias", which appear to be more commonly used.

RESPONSE

Thank you very much for your comment.
We used the term "prediction from false hidden feature" instead of "innovent bias".

We chose to use this particular phrase to describe the following possible error. For example, if patients diagnosed with cancer happened to wear red clothes and patients with benign lesions happened to wear blue clothes, the algorithm may predict their diagnosis based on the color of clothes. This unintended error is attributable to the ability of algorithms to detect even the most trivial features present in the data used for training. It is very difficult for investigators to realize which hidden findings are responsible for this error.
Not only visual features but class imbalance of training datasets can also affect the algorithm's performance. If the algorithm was trained with a particularly large number of acne cases, its output may be skewed toward acne. The risk of internal validation lies in such circumstances, because we may observe an excellent 'top accuracy'.

On the other hand, "hindsight bias" or "leakage bias" can be regarded as the investigator's mistake (mishandling of images, contamination of datasets, or misinterpreting causality). The bias we tried to point out in the text refers to unintentional (=innocent) errors, which is difficult to prevent. We strongly agree with you that "innocent bias" may be misleading for the readers, and have replaced it with "prediction from false hidden feature".

LINES RELATED TO THIS ISSUE
Line 465

5. In Line 422, it might be clarified "In this study, skin cancer diagnosis using biopsied cases [from images] proved to be a difficult task...", if our interpretation is correct here.
RESPONSE
We deeply appreciate your comment. We have edited the relevant text.
LINES RELATED TO THIS ISSUE
Line 506

6. In Line 462, "Caucasians subjects" -> "Caucasian subjects"
RESPONSE
Thank you very much. Changes have been made accordingly. Instead of 'Caucasian', we used the term 'white'.

7. In Line 474, "of dermatologist" -> "of dermatologists"
RESPONSE
Thank you very much. Changes have been made accordingly.
LINES RELATED TO THIS ISSUE
Line 567

8. For the Edinburgh evaluation, it might be explicitly stated whether the model output was restricted to the 10 classes.
RESPONSE

We appreciate your query. For the Edinburgh evaluation, we used all 178 outputs. This statement has been included in our revised manuscript.

LINES RELATED TO THIS ISSUE

Supplement Line 124~125

Supplement Line 161

Supplement Line 171

9. The figures appear to exhibit image artefacts in the manuscript PDF, although the original TIF/TIFF images appear alright.

RESPONSE

Thank you for your comment. We have checked that the figures in the Supplement section are of adequate quality. There seems to be a loss in image resolution during the online conversion of our manuscript and figures into PDF.

Reviewer #3: While I am still disappointed about their restrictive data sharing policy, the authors substantially improved the manuscript in the revised version.

RESPONSE

We would like to thank you for your valuable review of our manuscript. Also, we apologize for not being able to share the entire data used in this study.

Unlike X-ray or dermoscopy images, clinical photographs used in this study contain personally identifiable information (PII) which depict unique features of the patients' intimate body areas and anatomical structure. This is why approval is required from the Institutional Review Board (IRB) to even perform internal testing with the clinical photographs.

Disclosure of these datasets to third parties requires, at the minimum, the approval of the IRB of Severance Hospital.

Even if the necessary approvals could unanimously be attained, we would have to address the legal and regulatory issues arising from the disclosure. Furthermore, there are likely to be broader societal and ethical ramifications, not least any public outcry from the stern advocates of right to privacy.

We would like to also point out that in review articles regarding the use of deep learning algorithm in dermatology (https://www.jmir.org/2018/10/e11936/, https://www.sciencedirect.com/science/article/abs/pii/S0022202X2031201X), only 'a few' datasets used in published papers are available to the public.

Although we are not able to accommodate your request for the datasets for the reasons above, we understand that the test dataset needs to be shared among investigators to allow comparison between studies. That is why we carried out additional validation using the Edinburgh dataset, which is accessible to all.

Regarding data availability, we have added in our manuscript that we may provide the model in the form of API (application programming interface) if a large set of data is necessary for additional validation.

However, we regret to say that disclosure of the model is not possible for the following reasons:

1) Possibility for release of 'copy models' after slight modification or fine-tuning of our model
2) Possible commercial use of our model without permission. We have not yet made the offline model available for the public because it portends a risk of unrestricted and unauthorized commercialization.

In contrast to models dealing with other medical data such as radiologic images that can only be employed by medical professionals, our model can easily be commercially released to the public in the form of smartphone applications, for example.

To the best of our knowledge, two of our papers published in medical journals (http://www.jidonline.org/article/S0022-202X(18)30111-8/fulltext ,

https://doi.org/10.1371/journal.pone.0191493) are the only ones where the model used in the study was made available to the public. We believe that the models used in other papers (*nature*; https://www.nature.com/articles/nature21056, *nature medicine*; https://www.nature.com/articles/s41591-020-0842-3) were not made public for the same reasons mentioned above.
We do however understand that model disclosure is essential to demonstrate reproducibility of our results. Therefore, we have made a real-time web-demo available (http://modelderm.com , http://rcnn.modelderm.com) since 2018.
Most practicing dermatologists may not be familiar with direct model handling. Thus, we believe that a web-demo of our model is more suitable than releasing the model file itself. Whereas the model we posted online in 2017 (https://figshare.com/articles/software/Caffemodel_files_and_Python_Examples/5406223) was downloaded only 141 times, the web-demo is being actively tested (a daily average of 30 times). There also have been academic letters discussing its performance (https://pubmed.ncbi.nlm.nih.gov/29864435/ , https://pubmed.ncbi.nlm.nih.gov/29864434/).

LINES RELATED TO THIS ISSUE
Line 592~593
