# Supplementary material for: Assessment of deep neural networks for the diagnosis of benign and malignant skin neoplasms in comparison with dermatologists: A retrospective validation study
Source: PLoS Med. 2020 Nov 25;17(11):e1003381. doi: 10.1371/journal.pmed.1003381 (PMC7688128; doi:10.1371/journal.pmed.1003381)
Supplement: Attachment — Submitted filename: ResponseLetter_R4.pdf [file pmed.1003381.s011.pdf]


Requests from Editors:

Title : please add “a” in the title descriptor to change to “a retrospective validation study”

RESPONSE

Thank you very much for your review. As suggested, the title has been edited accordingly.

Abstract line 92- instead of “predictions” do you mean to say “predictive diagnoses” perhaps?

RESPONSE

We appreciate your comment. Yes, we meant to address “predictive diagnoses”. The sentence has been edited accordingly.

Please place full stops after the reference brackets

RESPONSE

Thank you for your valuable comment. We have placed full stops after each reference bracket.

Please copyedit the author summary section for English language and usage. Please also add bullet points to the summary

RESPONSE

Thank you for this query. The Author Summary section has been edited for language and usage. Also, bullet points have been added to our revised manuscript.
; Line 89~124

Figure 1: please revise the box “age <20 for IRB issue” to clearly indicate what the issue is

RESPONSE

We truly appreciate your comment. The IRB restricted enrollment of minors (<20 years of age) in the absence of an informed consent signed by parents or guardians, and the consents could not be waived even for retrospective analysis. Thus, we excluded patients under the age of 20. We clarified this point in our revised version of Figure 1 and its legend.
; Line 172~175, Line 563~564

As stated in previous decision letters, it is important for the methods section to state whether analyses were planned or not, regardless of whether the study was prospective or retrospective. Please state in the methods that there was no prospective analysis plan for this study, as you have indicated in the previous response to our request.

RESPONSE

We would like to thank you for this query. The following sentence has been added in the Methods section of our revised manuscript: “there was no prospective analysis plan for this study”

; Line 161

(https://rcnn.modelderm.com) – as mentioned in a couple of places as developed in their previous study – the link is broken. Please provide a working link

RESPONSE
We apologize for this issue. Recently, the website was temporarily inaccessible due to technical issues regarding SSL / TLS Certificate. This issue has been resolved and we checked that it is now accessible.

Line 489 – “To prevent data leakage” – please clarify?

RESPONSE
Thank you for your comment.
By “data leakage”, we meant to refer to train-test contamination of data (please refer to line 440 of our manuscript). For appropriate validation, datasets used for training and testing should be mutually exclusive. This means that there should be no case included in both training and test datasets.
; Line 472~473

Line 556 – “We expect that algorithm-based cancer screening will facilitate appropriate referrals.” - instead, use ...the algorithm may…

RESPONSE
We appreciate your valuable suggestion. The sentence has been edited accordingly.
; Line 535

Line 551 – “The algorithm can operate endlessly with minimal cost” should be removed as there is no cost analysis in this study

RESPONSE
Thank you for your comment. We removed the phrase you mentioned.

Line 572 – “patient metadata, should be exploited “ perhaps exploit isn’t the wisest of words here and 'should' can go also in place of 'may'

RESPONSE
We appreciate your kind comment. The sentence has been edited as suggested.
; Line 551

Please remove the filled consent form for privacy reasons and include a blank consent form for publication in the original language and an English translation.

RESPONSE
Thank you for your query. We hereby submit the black consent form (for in Korean and English) along with our revised manuscript.

STARD checklist- please use sections and paragraphs instead of page numbers as these will change during publication

RESPONSE
We deeply appreciate your query. We edited the STARD checklist using sections and paragraphs.

Please submit your algorithm and API in a public repository such as GitHub or Sourceforge or similar. In line with PLOS data policies, we ask that all methods, software used in studies such as these are provided without restriction. Please provide the URL and or accession number within the data statement. The restrictions on clinical images are acceptable.

RESPONSE
Thank you very much for your comment.
We have uploaded our algorithm and API on GitHub. The API can be used without restriction. In the Data Availability section, we have added comments on this issue.
The API is currently available for all (https://github.com/whria78/modelderm_rcnn_api)

Comments from Reviewers:
Any attachments provided with reviews can be seen via the following link:
****

In compliance with data protection regulations, you may request that we remove your personal registration details at any time. (Use the following URL: https://www.editorialmanager.com/pmedicine/login.asp?a=r). Please contact the publication office if you have any questions.
